# The Clinical Relevance and Tumor Promoting Function of C19orf10 in Kidney Renal Clear Cell Carcinoma

**DOI:** 10.3389/fonc.2021.725959

**Published:** 2021-09-06

**Authors:** Yanxin Lu, Ximian Liao, Tongyu Wang, Xiaowei Hong, Zesong Li

**Affiliations:** ^1^Guangdong Provincial Key Laboratory of Systems Biology and Synthetic Biology for Urogenital Tumors, Department of Urology, The First Affiliated Hospital of Shenzhen University, Shenzhen Second People’s Hospital (Shenzhen Institute of Translational Medicine), Shenzhen, China; ^2^Basic Medical Science Department, Zunyi Medical University, Zhuhai, China; ^3^Shenzhen Institute of Advanced Technology, Chinese Academy of Science, Shenzhen, China; ^4^Shenzhen Key Laboratory of Genitourinary Tumor, Department of Urology, The First Affiliated Hospital of Shenzhen University, Shenzhen Second People’s Hospital (Shenzhen Institute of Translational Medicine), Shenzhen, China; ^5^Neurosurgery Department, Peking University Shenzhen Hospital, Shenzhen, China

**Keywords:** kidney renal clear cell carcinoma, C19orf10, diagnostic biomarker, prognostic biomarker, PTEN/Akt, ZO-1

## Abstract

Kidney renal clear cell carcinoma (KIRC) is the most common primary renal neoplasms. Currently, there are few molecular indicators and therapeutic targets that can be used in diagnostic and prognostic assessment. In this study, we identified the C19orf10 expression in KIRC specimens and explored the diagnostic and prognostic value of C19orf10 in KIRC using TCGA and CPTAC database. Loss-of- and gain-of- function of C19orf10 was performed to investigate the roles of C19orf10 on KIRC cell viability, proliferation, migration and invasion *via* CCK-8, Edu incorporation and Transwell assays respectively. C19orf10 was overexpressed in KIRC tissues and the elevated C19orf10 expression was closely associated with clinicopathological characteristics of KIRC including histological grade, TNM stage, metastatic status. Silencing C19orf10 significantly suppressed the viability, proliferation, migration and invasion ability, while overexpression of C19orf10 promoted the progression and malignant phenotype in KIRC cells. Furthermore, C19orf10 exerted its carcinogenic function by regulating ZO-1 and PTEN/Akt signaling pathway. Moreover, the Kaplan–Meier survival analysis, Cox regression analysis and receiver operating curve analysis showed that patients with C19orf10 overexpression have poor survival time. C19orf10 could discriminate KIRC patients with high-risk from low-risk. Taken together, C19orf10 contributes to KIRC development *via* ZO-1 and PTEN/Akt signaling pathway and C19orf10 could serve as a potential diagnostic and prognostic candidate and therapeutic target of KIRC.

## Introduction

Kidney renal clear cell carcinoma (KIRC) is the most common primary renal neoplasms and accounts for 2%-3% of all adult malignancies (1, 2). Approximately 90% of KIRC cases are diagnosed incidentally on imaging detection in advanced stage due to most patients having no classic symptoms such as hematuria, flank pain and palpable masses (3, 4). In terms of clinical diagnosis and prognosis assessment, there are currently few molecular indicators and therapeutic targets that can be used in KIRC (5). Therefore, exploring new and effective molecules for the diagnosis, prognosis and treatment of KIRC is a pressing need.

C19orf10 (chromosome 19 open reading frame 10), also named myeloid-derived growth factor (MYDGF), was first identified in fibroblast-like synoviocytes cells and mainly located in the endoplasmic reticulum and Golgi apparatus (6, 7). Studies have found that C19orf10 which is made by bone marrow-derived monocytes and macrophages could protect cardiac myocytes and the heart after MI (8, 9). We analyzed our previously deep transcription sequencing data performed in 10 paired KIRC samples and found that the expression of C19orf10 was elevated in KIRC tissue compared with in para-carcinoma tissues (10). Recently, it has been demonstrated that C19orf10 was over-expressed in hepatocellular carcinoma and could enhance hepatocellular carcinoma (HCC) cell proliferation through Akt/mitogen signaling pathway (11). However, the diagnosis and prognostic values of C19orf10 in KIRC have not been explored and the functions of C19orf10 in the development of KIRC are unclear. This study aimed to evaluate the correlation between C19orf10 and clinical pathology and prognosis of KIRC patients. We also investigated the function of C19orf10 in KIRC. We found that C19orf10 expression was upregulated in specimens acquired from KIRC patients and both in The Cancer Genome Atlas (TCGA) and The National Cancer Institute’s Clinical Proteomic Tumor Analysis Consortium (CPTAC) dataset. High level of C19orf10 is associated with poor over-all survival and disease-free survival (DFS) rates in KIRC patients. C19orf10 silencing significantly suppresses KIRC cell growth as well as cell migration and invasion. Overexpression of C19orf10 promotes the proliferation, migration and invasion of KIRC cells. Furthermore, Zonula Occludens-1 (ZO-1) and phosphatase and tensin homolog deleted on chromosome ten (PTEN)/Akt pathway was involved in C19orf10-mediated KIRC cell survival and progression. Our study demonstrated that C19orf10 could be a candidate independent diagnostic and prognostic biomarker in KIRC and it is a potential tumor oncogene which can be used as a therapeutic target in KIRC.

## Materials and Methods

### Tissue Samples and Data Analysis

Specimens from KIRC patients were used in this study. This study included 30 samples from KIRC patients who were pathologically diagnosed with KIRC. No patients had received chemotherapy, radiotherapy or immunotherapy before surgery. Fresh samples were frozen promptly in liquid nitrogen and stored at -80°C. The research protocol was approved by the Ethics Committee of Shenzhen Second People’s Hospital (Shenzhen, China). The RNA expression data of C19orf10 was profiled based on normalized RNA-seq expression data sets of KIRC in TCGA database (TCGA_KIRC_exp_HiSeqV2-2015-02-24) from the UCSC Xena Browser (https://xenabrowser.net/datapages/). The protein expression of C9orf10 was profiled based on normalized proteomic analysis data in the CPTAC (Clinical Proteomic Tumor Analysis Consortium) database (https://proteomics.cancer.gov/data-portal). Differentially expressed C19orf10 in 10 paired KIRC tissues were obtained from previously reported data (10).

### Cell Culture

The human renal cell carcinoma cell lines ACHN, 769-P, OS-RC-2, 786-O, Caki-1 and Caki-2 cells and the normal renal proximal tubular epithelial cell line HK-2 were obtained from the American Type Culture Collection (Manassas, VA, USA). The human renal cell carcinoma cells were cultured in MEM (Gibco; Thermo Fisher Scientific, Inc.) or RPMI 1640 (Gibco; Thermo Fisher Scientific, Inc.) with 10% FBS (Gibco; Thermo Fisher Scientific. Inc.), 100 U/ml penicillin (Gibco; Thermo Fisher Scientific, Inc.) and 100 µg/ml streptomycin (Gibco; Thermo Fisher Scientific, Inc.). The human renal proximal tubular cell line (HK-2), as a control cell line, was cultured in keratinocyte serum-free medium (K-SFM) with 0.05 mg/ml bovine pituitary extract (BPE) and 5 ng/ml human recombinant epidermal growth factor (EGF). Cells were grown at 37°C in a 5% CO_2_ atmosphere.

### SiRNAs and Plasmids Transfection

The pCMV6-Entry-C19orf10 or pCMV6-Entry plasmids were purchased from OriGene Technologies, Inc. The siRNA sequences were as follows: siR-C19orf10-1, TGTCCAAGCTGGTGATTGT; siR-C19orf10-2, CCCTCTGAAAACTGAGGAA. All siRNAs and siR-NC were synthesized in Guangzhou RiboBio Co., Ltd.

The plasmids or siRNAs were transfected using Lipofectamine 3000 or RNAimax (Invitrogen, Carlsbad, CA, USA) according to the manufacturer’s instructions. The final concentration of siRNA was 50 nM.

### RT-qPCR

Total RNA was extracted using TRIzol reagent (Invitrogen; Thermo Fisher Scientific, Inc.) and synthesized cDNA using PrimeScript™ RT reagent with gDNA Eraser (Takara Bio, Inc., Otsu, Japan). The mRNA levels of genes were analyzed using a SYBR Premix Pro Taq HS qPCR kit (Accurate Bio, Inc.) following the manufacturer’s instructions. The primer sequences were shown below: C19orf10, forward 5’-GGCGTCGTGCATTCCTTCT-3’, reverse 5’-CCATTGCTCATTGGTCCCTC-3’; PTEN, forward 5’-TGGATTCGACTTAGACTTGACCT-3’, reverse 5’-GGTGGGTTATGGTCTTCAAAAGG-3’; ZO-1, forward 5’-AGCCATTCCCGAAGGAGTTG-3’, reverse 5’-CAGCTCCACAGGCTTCAGG-3’; and ACTB, forward 5’-TGAAGATCAAGATCATTGCTCCTC-3’, reverse 5’-AACTAAGTCATAGTCCGCCTAGAAG-3’. qPCR was conducted on the ABI 7300 Real-Time PCR system (Applied Biosystems; Thermo Fisher Scientific, Inc.) and was calculated using the 2−ΔΔCq method as previously described (12). All reactions were performed in triplicate.

### Immunohistochemical staining

Human KIRC tissue microarray including 33 cases of paraffin-embedded tissues was purchased from Shanghai Outdo Biotech Co., Ltd. The expression of C19orf10 was assessed using immunohistochemistry (IHC). The sections were dewaxed in xylene and graded alcohols, and antigen was repaired with sodium citrate solution in a microwave. Then the tissues were treated by 3% hydrogen dioxide and blocked with 5% goat serum. The sections were incubated with antibody against C19orf10 (Proteintech Group Inc., Wuhan, China), followed by incubation with secondary antibody labeled with peroxidase and visualized with a DAB kit (Zhong Shan Golden Bridge Biotechnology, Beijing, China). The nucleus was stained with hematoxylin. The IHC score was obtained by multiplying the intensity score (0, negative; 1, weak; 2, moderate; 3, strong) by the score for the percentage of positively stained cells (1, ≤25%; 2, 26%-50%; 3, 51%-75%; 4, >76% of positively stained cells).

### Western Blotting

Western blotting was carried out as described previously (13). The protein was extracted from the cells using RIPA lysis buffer with protease inhibitor cocktail (Roche), and the concentration was measured using the Poerce BCA Protein Assay Kit (Thermo Fisher Scientific, Inc.). Proteins were separated by SDS-PAGE, transferred onto PVDF membranes and subsequently blocked in 5% skimmed milk. Next, the membranes were incubated with primary antibodies and secondary antibodies in order. Bands were visualized using ECL kit (EMD Millipore) and detected by the Alliance Imaging system (Uvitec, Cambridge, UK).

The following antibodies were used: C19orf10 (1:1000, PROTEINTECH, Inc), β-tubulin (1:5000, Abcam, Cambridge, UK), PTEN (1:1000, Cell Signaling Technology, Inc.), ZO-1 (1:1000, Cell Signaling Technology, Inc.), Akt (1:1000, ZENBIO.), phospho-Akt (Ser473) (1:1000, Cell Signaling Technology, Inc.), phospho-Akt (Thr308) (1:1000, Cell Signaling Technology, Inc.), goat anti-rabbit (1:1,000, cat no. sc-2004, Santa Cruz Biotechnology, Inc.), horse anti-mouse (1:1,000, cat no. 7076P2, Cell Signaling Technology, Inc.).

### Cell Viability Assay

The viability of ACHN and 769-P cells was measured by the cell counting kit-8 (CCK-8; Dojindo, Kumamoto, Japan). A total of 1500 cells per well were seeded into 96 well plates. After treatment, 10 μL CCK8 regent was added into 90 μL cell culture medium with 10% FBS and cells were incubated at 37°C for 2 hours. The light absorbance was measured at 450 nm using a microplate reader.

### EdU Incorporation Assay

The proliferation of cells was detected using the EdU incorporation assay kit (RiboBio, China) according to the manufacturer’s instructions. The C19orf10 knockdown or overexpression cells were seeded in 96-well plates at 5×10^3^ cells per well. The images were taken at 567 nm excitation using a fluorescent microscope.

### Cell Migration and Invasion Assay

We detected cell migration and invasion using Transwell assay as described previously (14). In total, 5×10^4^ cells/well in serum-free medium were seeded into the top chamber (pore size, 8 µm; Corning Incorporated, Corning, NY, USA). For the cell invasion assay, the upper chamber was coated with Matrigel (1:8; 50 µl/well; BD Biosciences, San Jose, CA, USA) and the cell number is 8×10^4^ cells/well for 769-P. The bottom chamber was filled with conditioned medium with 10% FBS. After 24 h incubation, the cells were fixed with 4% paraformaldehyde and stained using 0.1% crystal violet for 5 min at room temperature. Images were captured using an inverted microscope.

### Statistical Analysis

Statistical analyses were performed using GraphPad Prism 8.0 (La Jolla, CA, USA) and SPSS 23.0 (SPSS Inc., Chicago, IL, USA). All values are expressed as the mean ± SD. The differences between groups were indicated in figures and were calculated by t-test for two groups or ANOVA for more than two groups. Survival curves were analyzed using the Kaplan–Meier method and significance was estimated using log-rank tests. univariate and multivariate Cox regression analyses were used to evaluate the correlation of different factors including C190rf10 expression on disease free survival. Diagnostic potential of C19orf10 was performed using the area under the curve (AUC) and 95% confidence interval (CI) in receiver operating curve (ROC) analysis. P<0.05 was considered statistically significant.

## Results

### C19orf10 Expression Levels Elevated in KIRC and Correlated With Clinical Characteristics

**Figure 1:**
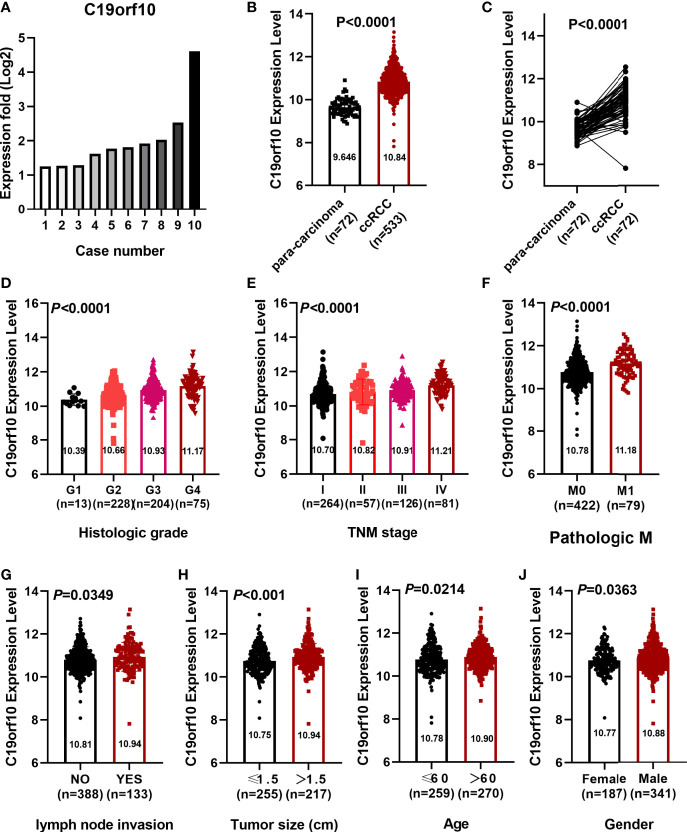
C19orf10 expression elevated in KIRC and correlated with clinical characteristics. **(A)** C19orf10 expression in 10 paired KIRC samples. **(B)** C19orf10 expression in KIRC in TCGA dataset. **(C)** C19orf10 expression in 72 paired KIRC in TCGA dataset. **(D–J)** Relative mRNA expression level of C19orf10 in TCGA dataset with differential histologic grade **(D)**, TNM stage **(E)**, pathologic metastasis **(F)**, lymph node invasion **(G)**, tumor size **(H)**, age **(I)** and gender **(J)**. The numbers of samples are indicated in figures, Mean ± SD is shown. *P* values are calculated by Student’s t-test or one-way ANOVA and indicated in figures.

Our previously deep transcription sequencing data performed in 10 paired KIRC samples was analyzed in detail (10) and the results showed that KIRC tissues had higher C19orf10 expression levels than para-carcinoma tissues (**Figure 1A**). This gives us the first hint that it is essential to further investigate the potential values and functions of C19orf10 in KIRC progression. TCGA cancer databases and CPTAC dataset were used to evaluate the expression level of C19orf10 in KIRC and adjacent normal tissues. We first assessed the expression levels of C19orf10 in KIRC tumor and normal tissues in TCGA dataset. The results showed that C19orf10 expressions in KIRC were significantly higher than adjacent non-tumor tissues (**Figure 1B**). Moreover, we analyzed C19orf10 mRNA levels in 72 pairs of KIRC and corresponding para-carcinoma kidney tissues in TCGA dataset. As **Figure 1C** revealed, C19orf10 was significantly upregulated in KIRC tissues. To investigate the clinical significance of C19orf10 expression in KIRC, we analyzed TCGA dataset and results showed that the expression of C19orf10 was positively associated with advanced histological grade (**Figure 1D**) and TNM stage (**Figure 1E**). Additionally, the results also showed that C19orf10 was expressed higher in metastatic tissues than non-metastatic tissues (**Figure 1F**) and the expression of C19orf10 in lymph nodes with tumor metastasis was higher than the tissues without metastasis (**Figure 1G**). Meanwhile, C19orf10 was overexpressed in bigger size tumor, older patients (age>60) and male patients in TCGA dataset (**Figures 1H–J**).

**Figure 2:**
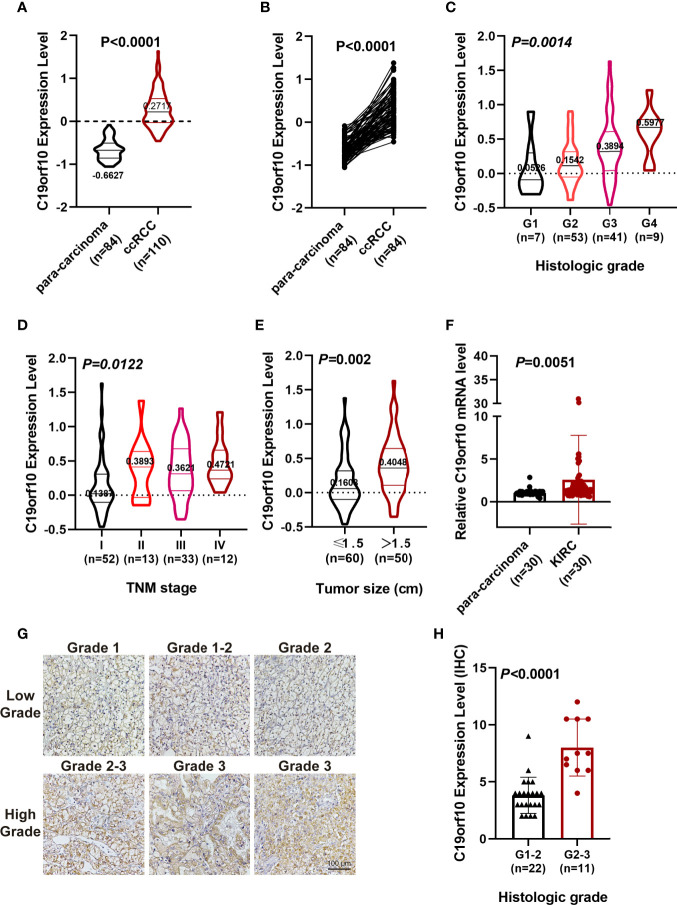
C19orf10 protein up-regulated in CPTAC database and associates with KIRC clinical pathology. **(A)** C19orf10 protein expression in KIRC in CPTAC dataset. **(B)** C19orf10 protein expression in 84 paired KIRC in CPTAC dataset. **(C–E)** Relative protein expression level of C19orf10 in CPTAC dataset with differential histologic grade **(C)**, TNM stage **(D)** and tumor size **(E)**. **(F)** C19orf10 expression in 30 paired KIRC samples by qPCR detection, n=30. **(G)** Representative immunohistochemical images of C19orf10 in differential histologic grade in KIRC tissues. Scale bar, 100 μm. **(H)** Elevated C19orf10 expression in high grade KIRC tissues (grade 2-3, grade 3) compared with low grade ones (grade 1, grade 1-2 and grade 2). The numbers of samples are indicated in figures, Mean ± SD is shown. *P* values are calculated by Student’s t-test or one-way ANOVA and indicated in figures.

Furthermore, CPTAC dataset showed a similar phenomenon that the protein level of C19orf10 was highly expressed in the KIRC tissues (**Figure 2A**). The analysis of C19orf10 expression level in 84 pairs of KIRC and matched para-carcinoma tissues revealed that C19orf10 protein was significantly higher in tumor compared to normal tissues (**Figure 2B**). In consistent with the TCGA database, the protein expression level of C19orf10 was high in the KIRC tissues with higher tumor grade (**Figure 2C**), advanced TNM stages (**Figure 2D**) and larger tumor size (**Figure 2E**). However, the correlation between C19orf10 protein expression and the age and gender of patients was not statistically significant (data not shown).

To validate our sequencing and database analysis results, we performed qPCR to detect C19orf10 mRNA expression level in 30 paired KIRC tissues and adjacent noncarcinoma tissues. The expression of C19orf10 was enhanced in KIRC tissues (**Figure 2F**). Furthermore, the KIRC tissues of 33 patients were collected and processed with immunohistochemical staining by C19orf10 antibody, including 22 cases of grade 1-2 (including grade 2, low grade) and 11 cases of grade 2-3 (high grade). When the expression level of C19orf10 was compared with differential histologic grade in 33 KIRC cases, we observed an increased C19orf10 expression as the KIRC histological grade progressed (**Figures 2G, H**).

These results demonstrated that C19orf10 was overexpressed in KIRC tissues, suggesting that C19orf10 play important roles in KIRC progression.

### Silencing C19orf10 Reduced KIRC Cell Viability and Proliferation *In Vitro*

**Figure 3:**
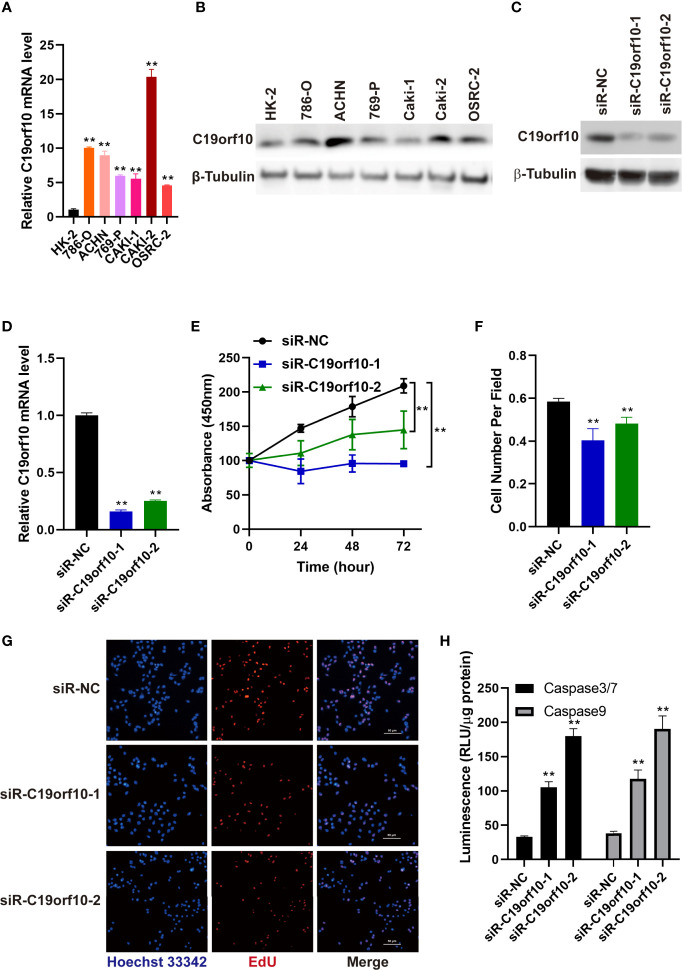
Silencing C19orf10 reduced KIRC cell viability and proliferation *in vitro*. **(A)** C19orf10 mRNA expression in KIRC cell lines and human renal proximal tubular cells (HK-2), n=3. **(B)** C19orf10 protein expression level in KIRC cell lines and human renal proximal tubular cells. **(C, D)** The silencing efficiency of siRNAs targeting C19orf10 on protein **(C)** and mRNA level **(D)**, n=3. **(E)** Suppression of C19orf10 inhibited the KIRC cell viability in CCK-8 assay, n=3. **(F, G)** Knockdown of C19orf10 suppressed KIRC cell proliferation in EdU incorporation assay, n=8. **(H)** Knockdown of C19orf10 induced more cell apoptosis in ACHN cells, n=3. Scale bar, 50 μm. Mean ± SD is shown. **P < 0.01 by Student’s t-test and two-way ANOVA.

We detected the mRNA and protein level of C19orf10 in KIRC cell lines and human renal proximal tubular cell (HK-2, as a normal control cell) using RT-qPCR and western blotting. The results showed that the expression of C19orf10 was elevated in KIRC cells (**Figures 3A, B**).

To explore the oncogenic function of C19orf10 in KIRC, we silenced C19orf10 expression in ACHN cells which have a high level of C19orf10 by two individual siRNAs. The silencing efficiency was verified by qPCR and Western blotting. The results showed that the levels of C19orf10 decreased at least 70% using two independent siRNAs (**Figures 3C, D**). Furthermore, we found that suppression of C19orf10 significantly inhibited the cell viability in CCK-8 assay (**Figure 3E**). The EdU incorporation assay revealed that silencing C19orf10 resulted in a decrease of cell proliferation, as well as less EdU positive cells in two C19orf10 knockdown cells compared with the control cells (siR-NC) (**Figures 3F, G**). Moreover, compared with the control siRNAs, siR-C19orf10-1 and siR-C19orf10-2 induced more cleaved caspase 3/7 in KIRC cells which indicated that knocking down C19orf10 elevated apoptosis of KIRC cells (**Figure 3H**). Collectively, these results indicate that silencing C19orf10 inhibits KIRC cell proliferation and enhances cell apoptosis.

### C19orf10 Enhances the Viability and Proliferation of KIRC Cells

**Figure 4:**
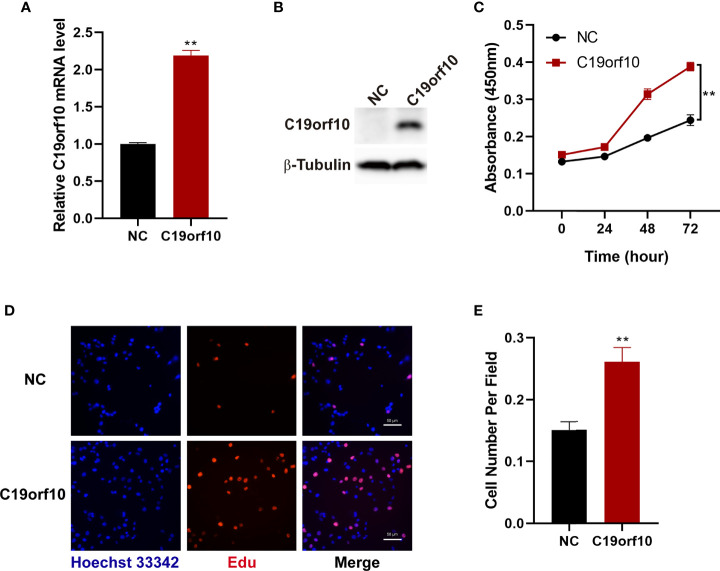
Overexpression of C19orf10 enhances the viability and proliferation of KIRC cells. **(A, B)** The verify of C19orf10 overexpression on mRNA **(A)** and protein level **(B)**, n=3. **(C)** C19orf10 promoted the viability of 769-P cells, n=3. **(D, E)** C19orf10 increased the proliferation of 769-P cell, n=8. Scale bar, 50 μm, Mean ± SD is shown, **P < 0.01 by two-way ANOVA **(C)**, Student’s t-test **(A, E)**.

To further investigate the function of C19orf10 in KIRC cells, we overexpressed C19orf10 in 769-P cells that have low level of C19orf10 *via* transfected with pCMV6-entry-C19orf10, the pCMV6-entry empty vector as negative control (NC). To validate the C19orf10 expression, we performed qPCR and Western blotting to detect C19orf10 expression level in 769-P cells. As shown in **Figures 4A, B**, the C19orf10 expression was higher in 769-P cells after transfection with pCMV6-entry-C19orf10 compared with control cells. Contrary to knocking down KIRC cells, C19orf10 over-expressed cells showed higher cell viability than control cells in CCK-8 assay (**Figure 4C**). Compared with the control group, the Edu incorporation assay showed that C19orf10 significantly increased cell proliferation (**Figures 4D, E**). In summary, C19orf10 plays a crucial role in KIRC cell proliferation *in vitro*.

### C19orf10 Promotes the Migration and Invasion of KIRC Cells

**Figure 5:**
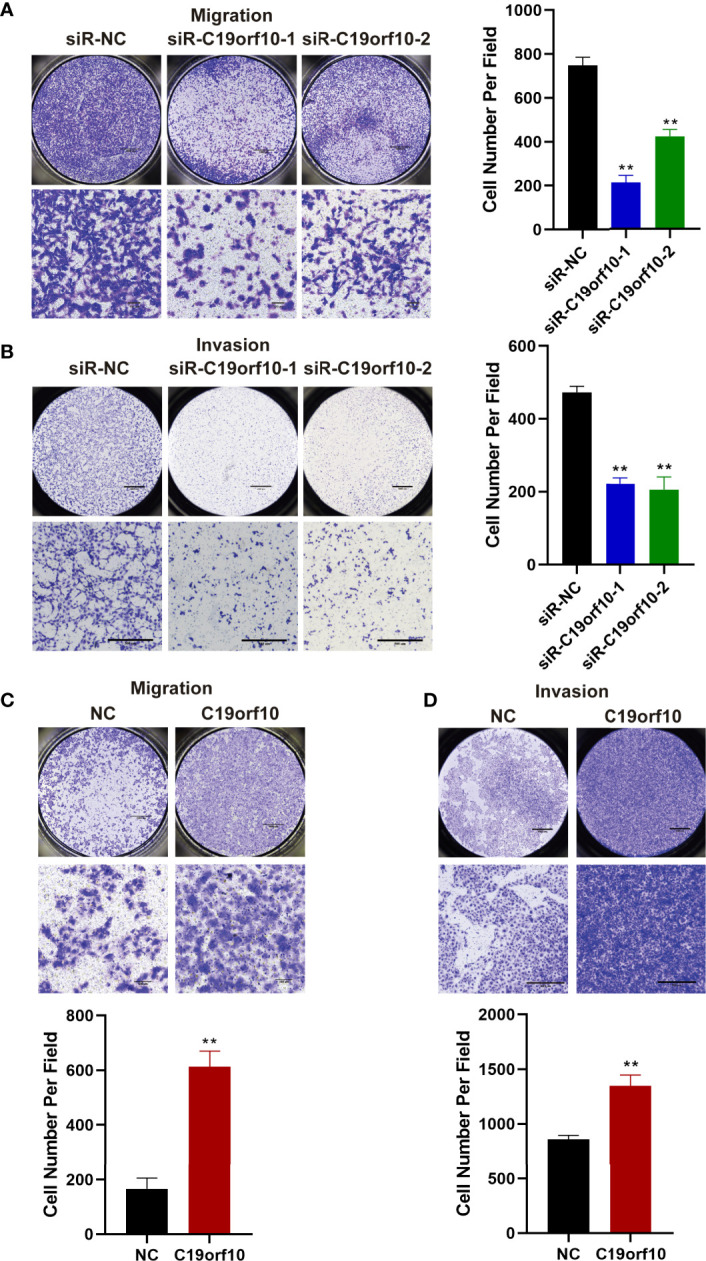
C19orf10 promotes the migration and invasion of KIRC cells. **(A, B)** Silencing C19orf10 inhibited the migration and invasion ability of ACHN cell in transwell migration and invasion assay. **(C, D)** C19orf10 promoted the migration and invasion of 769-P cell. Scale bar, 1000 μm for overall images, 100 μm for partial migration pictures, 500 μm for partial invasion pictures. Mean ± SD is shown, n = 5. **P < 0.01 by Student’s t-test.

The above observations showed that the expression of C19orf10 was positively associated with KIRC advanced histological grade and TNM stage. Additionally, the C19orf10 level was positively correlated with lymph node and histological metastasis of KIRC patients. Therefore, we sought to explore whether C19orf10 was involved in regulating cell migration and invasion of KIRC using Transwell assay without or with Matrigel. Knocking down endogenous C19orf10 in ACHN cells using siRNAs significantly suppressed the cell migration and invasion ability. In ACHN cells, silencing C19orf10 significantly decreased the number of cells passing through the polycarbonate membrane and Matrigel (**Figures 5A, B**). Conversely, C19orf10 over-expressed cells exhibited greater migration and invasion ability in Matrigel uncoated and coated Transwell assay respectively. The results showed more cells passing though the membrane and Matrigel in C19orf10 overexpression group (**Figures 5C, D**). These results indicate that C19orf10 is potentially involved in KIRC cell migration and invasion.

### C19orf10 Promotes KIRC Progression *via* ZO-1 and PTEN/Akt Pathway

**Figure 6:**
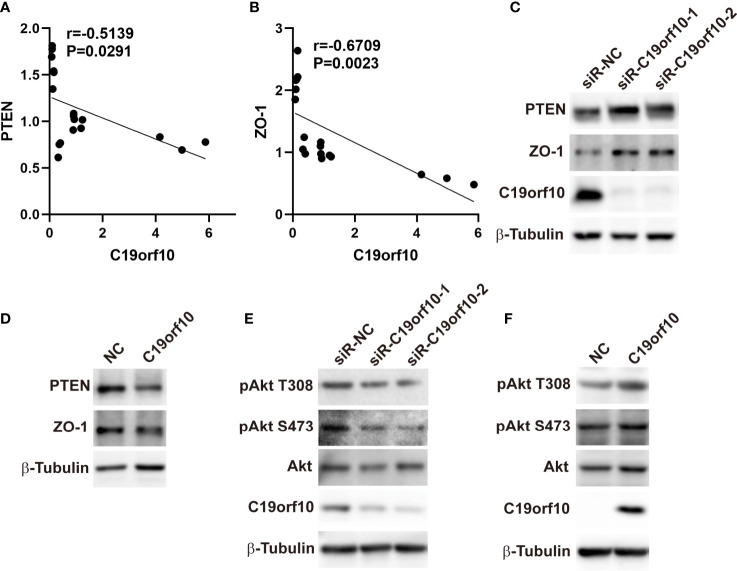
C19orf10 promotes KIRC progression *via* ZO-1 and PTEN/Akt pathway. **(A)** ZO-1 was the C19orf10 negatively regulated genes. (Pearson correlation analysis, n=18). **(B)** PTEN was negatively correlated with C19orf10. (Pearson correlation analysis, n=18). **(C)** Knockdown of C19orf10 increased PTEN and ZO-1 expression in ACHN cells. **(D)** Overexpression of C19orf10 decreased PTEN and ZO-1 expression in 769-P cells. **(E)** Knockdown of C19orf10 inhibited the phosphorylation of Akt. **(F)** Overexpression of C19orf10 activated Akt.

To explore the molecular mechanism of C19orf10 in promoting the proliferation and malignant of KIRC cells, we performed qPCR to identify the differentially expressed genes and high correlation genes in C19orf10 silencing and overexpression cells. The results showed that ZO-1 and PTEN were C19orf10 negative regulated genes (**Figures 6A, B**). Furthermore, in consistent with the altered mRNA expression, the knocking down of C19orf10 increased the protein levels of PTEN and ZO-1 (**Figure 6C**), as well as overexpression of C19orf10 decreased the PTEN and ZO-1 protein level (**Figure 6D**). Akt is a well-known downstream target of PTEN, therefore, we hypothesized that C19orf10 may potential active Akt *via* downregulation of PTEN. Subsequently, we evaluated the phosphorylation level of Akt in C19orf10 silencing and overexpressing KIRC cells. As shown in **Figure 6E**, the inhibition of C19orf10 significantly inactivated Akt. Consistently, the ectopic expression of C19orf10 increased the phosphorylation of Akt (**Figure 6F**). Thus, our data indicated that PTEN/Akt and ZO-1 are involved in C19orf10 mediated KIRC progression.

### C19orf10 Is a Potential Prognostic and Diagnostic Biomarker in KIRC

**Figure 7:**
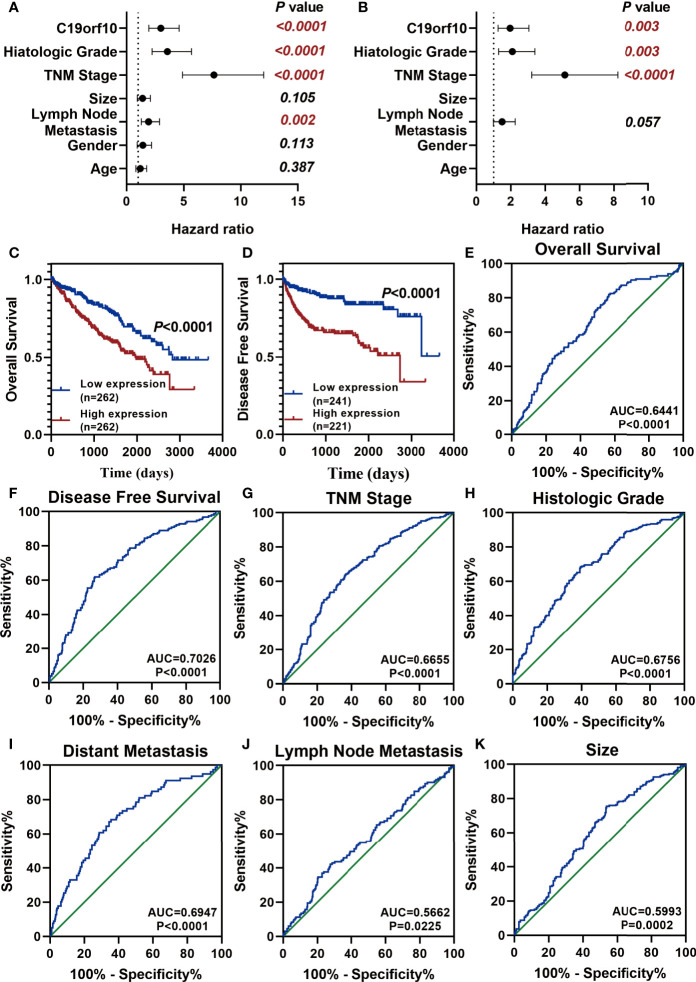
C19orf10 is a potential prognostic and diagnostic biomarker in KIRC. **(A, B)** Univariate Cox regression analysis **(A)** and multivariate Cox regression analysis **(B)** of the hazard ratios revealed that increased C19orf10 could be an independent prognostic biomarker for the disease free survival in KIRC patients. **(C)** Kaplan-Meier overall survival curve of KIRC patients with low (*n* = 262) and high (*n* =262) C19orf10 expression in TCGA dataset. **(D)** Kaplan-Meier analysis of disease free survival in KIRC patients based on the expression level of C19orf10, low (*n* = 241) and high (*n* =221). **(E–K)** ROC curve analysis of overall survival **(E)**, disease free survival **(F)**, TNM stage **(G)**, histologic grade **(H)**, distant metastasis **(I)**, lymph node metastasis **(J)**, tumor size **(K)** for C19orf10 as a biomarker to predict the KIRC prognosis and discriminate KIRC patients with high risk from low risk. The calculated AUC and P values are reported in figures.

After analyzing the expression and function of C19orf10 in KIRC, we evaluated if it is a candidate prognostic and diagnostic biomarker of KIRC patients. We performed Cox regression analysis to explore whether C19orf10 is an independent prognostic predictor of KIRC patients in TCGA dataset. Univariate Cox regression analysis revealed that C19orf10, histologic grade, TNM stage, lymph node metastasis was significantly correlated with the disease-free survival (DFS) of KIRC patients (**Figure 7A** and **Supplementary Table S1**). Multivariate Cox regression analysis illustrated that C19orf10 could be an independent factor to predict the prognosis of KIRC patients (**Figure 7B** and **Supplementary Table S1**). Kaplan-Meier survival analysis of C19orf10 in TCGA dataset revealed that higher C19orf10 expression level was significantly correlated with poor overall survival and disease-free survival in patients with KIRC (**Figures 7C, D**). Moreover, the ROC curve was performed to determine the diagnostic accuracy of C19orf10 in patients with KIRC. As showed in **Figures 7E–K** and **Supplementary Table S2**, the areas under the curve (AUCs) of C19orf10 for overall survival, disease free survival, TNM stage, histologic grade, distant metastasis, lymph node metastasis and tumor size was 0.6441, 0.7026, 0.6655, 0.6756, 0.6947, 0.5662, 0.5993 respectively. These results indicate that C19orf10 has high predictive accuracy for overall survival and disease free survival, as well as distinguishing the advanced TNM stages (SIII/SIV) from early stages (SI/SII), high histologic grades (G3/G4) from low histologic grades (G1/G2), with distant metastasis from without distant metastasis. These results suggested that C19orf10 could be an attractive prognostic factor for DFS and a potential diagnostic marker to discriminate KIRC patients with high-risk from low-risk.

## Discussion

KIRC accounts for 75% of histological subtype of renal cell carcinoma (RCC) and is the most lethal urological cancer (3, 15). Approximately 30% of patients are diagnosed as metastatic, and about one-third of patients showed recurrence after surgical resection (16). Therefore, precise diagnostic and prognostic factors and therapeutic targets are urgently needed (17). In the present study, we found that C19orf10 was highly expressed in KIRC and had an increased level with the development of KIRC. In addition, we explored the function of C19orf10 on KIRC cells and found that C19orf10 could promote cell viability, proliferation, migration and invasion. Multivariate analysis and survival analysis showed that C19orf10 was a potential diagnostic and prognostic factor in KIRC. Collectively, these results indicate that C19orf10 promotes the progression of KIRC and could be a diagnostic and an independent prognostic biomarker for KIRC progression.

C19orf10 is a 173-amino acid secreted protein and highly expressed in synovial fluids with arthropathies (6). C19orf10 has been shown to protect against cardiac injury by promoting cardiac myocyte survival and angiogenesis after myocardial infarction (8). Cardiac injury may cause increased secretion of C19orf10 which could improve heart regeneration by promoting cardiomyocyte proliferation through c-Myc/FoxM1 pathway in both adult and neonatal mouse hearts (9). Moreover, studies have also linked C19orf10 to nephrin expression and podocyte injury in diabetic kidney disease (18). Recently, two studies revealed that C19orf10 was upregulated in hepatocellular carcinoma (HCC) and affected the liver CSCs self-renewal ability (11, 19). However, the expression and function of C19orf10 in KIRC is elusive.

Our previous deep transcription sequencing data of 10 paired KIRC samples showed that C19orf10 expression level was elevated in KIRC tissue than in para-carcinoma tissues. Our study verified that C19orf10 was upregulated in 30 more KIRC tissues. TCGA and CPTAC database analysis revealed that the elevated mRNA and protein level of C19orf10 is closely related to the higher histologic grade, advanced TNM stage and distant metastasis of KIRC patients.

We further investigated the function of C19orf10 in KIRC cells using loss and gain of function strategies. Silencing C19orf10 suppressed the viability, proliferation, migration and invasion of KIRC cells, while overexpression of C19orf10 significantly promoted the progression of KIRC cells. To gain insight into the possible mechanism underlying C19orf10 roles in KIRC, we performed qPCR to identify the related functional genes in C19orf10 knockdown and overexpression cells. Considering the Akt related pathways closely correlates with C19orf10, in addition, epithelial-mesenchymal transition (EMT) and cell cycle related genes play a critical role in tumor progression. Hence, we designed the primers with PTEN, Axin1, Axin2, ZO-1, Vimentin, P21, P27 and P53 to screen C19orf10 associated genes using qPCR and validated by Western blotting. QPCR results showed that ZO-1, PTEN, P21 and P27 were negative regulated genes of C19orf10. There is no statistical significance for Vimentin, P53, Axin1 and Axin2 (data is not shown). However, the PTEN and ZO-1 expression was validated by western blotting. ZO-1 is an important tight junction molecule which is expressed at low level in various types of cancers to regulate the migration and invasion ability of cells (20, 21). Our findings first revealed that silencing C19orf10 led to elevated expression of ZO-1 while overexpression of C19orf10 reduced ZO-1 expression. It indicates that C19orf10 promotes the migration and invasion of KIRC cells *via* decreasing the tight connections between cells. PTEN is a tumor suppressor and regulates a wide range of essential biological processes including cell proliferation, cell survival, migration and metabolism through PI3K/Akt signaling pathway (22, 23). Many evidence indicated that Akt related pathways are indispensable for C19orf10-mediated cellular function. Recently, it has been demonstrated that C19orf10 could enhance HCC cell proliferation through Akt/mitogen signaling pathway (10). Another study also revealed that C19orf10 contributed to the activation of Akt/Bcl-2-associated cell death promoter signaling pathway in podocyte (18). C19orf10 activated the PKA/GSK-3β/β-catenin and MAPK/MEK/ERK pathways in diabetic mice and STC-1 cells (24). It has been reported that recombinant human C19orf10 could regulate human coronary artery endothelial cell proliferation and survival by MAPK/STAT3 and the cyclin D1 signaling pathway (25). So far the underlying mechanism of C19orf10 regulated Akt related pathways is unknown. Here, we observed that C19orf10 negatively regulated PTEN expression, then affected the phosphorylation of Akt, indicating that C19orf10 might regulate the Akt activity *via* PTEN. These results suggested that the effects of C19orf10 in KIRC may be mediated by PTEN/Akt and ZO-1.

There are limitations in this study. The roles and exact mechanisms of C19orf10 in KIRC require further *in vivo* and *in vitro* investigation. Data on the diagnostic and prognostic value were obtained from TCGA and CPTAC database. In the future, we will collect more clinical samples to verify the diagnostic and prognostic potential of C19orf10 in KIRC.

In conclusion, the C19orf10 mRNA and protein expression was upregulated and correlated with advanced clinical progression of KIRC tissues. In addition, the effect of C19orf10 in promoting the progression of KIRC cells is by regulating PTEN/Akt and ZO-1. Moreover, we proved that C19orf10 could be an attractive prognostic factor for DFS and a potential independent diagnostic marker to discriminate KIRC patients with high-risk from low-risk. These findings open up a new potential diagnostic and therapeutic strategy for KIRC.

## Data Availability Statement

The datasets presented in this study can be found in online repositories. The names of the repository/repositories and accession number(s) can be found in the article/**Supplementary Material**.

## Ethics Statement

The studies involving human participants were reviewed and approved by the Ethics Committee of Shenzhen Second People’s Hospital (Shenzhen, China). The patients/participants provided their written informed consent to participate in this study.

## Author Contributions

Conceptualization, Supervision, Writing - Original Draft, Methodology: YL. Resources, Investigation: XL. Investigation, Visualization: TW. Software, Validation, Resources: XH. Supervision, Project administration, Writing- Reviewing and Editing: ZL. All authors contributed to the article and approved the submitted version.

## Funding

This research was funded by The natural science foundation of Guangdong province (2020A1515010195); The Shenzhen Project of Science and Technology (JCYJ20190806163816220); The Program of Science and Technology Department of GuiZhou Province(Qian Ke He Ji Chu-ZK[2021]449); The China Postdoctoral Science Foundation (2020M672834); Guangdong Key Laboratory funds of Systems Biology and Synthetic Biology for Urogenital Tumors (2017B030301015); The Program of Science and Technology Department of GuiZhou Province (QKHPTRC-201905612) and The National Natural Science Foundation of China (81972366).

## Conflict of Interest

The authors declare that the research was conducted in the absence of any commercial or financial relationships that could be construed as a potential conflict of interest.

## Publisher’s Note

All claims expressed in this article are solely those of the authors and do not necessarily represent those of their affiliated organizations, or those of the publisher, the editors and the reviewers. Any product that may be evaluated in this article, or claim that may be made by its manufacturer, is not guaranteed or endorsed by the publisher.

## Supplementary Material

Supplementary Table 1 Univariate and multivariate Cox regression analyses of clinical factors for RFS survival in KIRC (TCGA dataset). Abbreviations: HR, hazard ratio; CI, confidence interval; TNM, Tumor node metastasis.

Supplementary Table 2 ROC curve analysis of the prognostic and diagnostic potential of C19orf10 in KIRC (TCGA dataset). Abbreviations: AUC, area under the curve; TNM, Tumor node metastasis.
