# Supplementary material for: The Clinical Relevance and Tumor Promoting Function of C19orf10 in Kidney Renal Clear Cell Carcinoma
Source: Front Oncol. 2021 Sep 6;11:725959. doi: 10.3389/fonc.2021.725959 (PMC8451477; doi:10.3389/fonc.2021.725959)
Supplement: Supplementary Table 1 — Univariate and multivariate Cox regression analyses of clinical factors for RFS survival in KIRC (TCGA dataset). Abbreviations: HR, hazard ratio; CI, confidence interval; TNM, Tumor node metastasis. [file Table_1.pdf]

**Table S1. Univariate and multivariate Cox regression analyses of clinical factors for RFS survival in KIRC (TCGA dataset).**

| Variables | Univariate analysis | | | Multivariate analysis | | |
|---|---|---|---|---|---|---|
| | HR | 95%CI | *P* value | HR | 95%CI | *P* value |
| **Gender (Male *vs.* Female)** | 1.418 | (0.921, 2.185) | 0.113 | | | |
| **Age (＞60 years *vs.*≤60 years )** | 1.189 | (0.803, 1.762) | .387 | | | |
| **Histologic Grade (G1/2-G3/4)** | 3.564 | (2.236, 5.679) | <0.0001 | 2.099 | (1.295, 3.402) | *0.003* |
| **TNM Stage (I/II-III/IV)** | 7.661 | (4.891, 12.001) | <0.0001 | 5.146 | (3.223, 8.214) | <0.0001 |
| **Lymph node metastasis (Yes *vs.* No)** | 1.918 | (1.277, 2.882) | 0.002 | 1.494 | (0.988, 2.259) | 0.057 |
| **Tumor size ( ＞1.5cm *vs.*≤1.5cm)** | 1.392 | (0.933, 2.077) | *0.105* | | | |
| **C19orf10 level** | 2.989 | (1.945, 4.593) | <0.0001 | 1.964 | (1.263, 3.054) | 0.003 |

**Abbreviations:** HR: hazard ratio, CI: confidence interval, TNM: Tumor node metastasis.
