# Supplementary material for: The Clinical Relevance and Tumor Promoting Function of C19orf10 in Kidney Renal Clear Cell Carcinoma
Source: Front Oncol. 2021 Sep 6;11:725959. doi: 10.3389/fonc.2021.725959 (PMC8451477; doi:10.3389/fonc.2021.725959)
Supplement: Supplementary Table 2 — ROC curve analysis of the prognostic and diagnostic potential of C19orf10 in KIRC (TCGA dataset). Abbreviations: AUC, area under the curve; TNM, Tumor node metastasis. [file Table_2.pdf]

Table S2. ROC curve analysis of the prognostic and diagnostic potential of C19orf10 in KIRC (TCGA dataset).

	AUC	95% CI	*P* value
**Overall Survival**	0.6441	(0.5941, 0.6940)	<0.0001
**Disease-Free Survival**	0.7026	(0.6538, 0.7514)	<0.0001
**Histologic Grade (G3-4 *vs.* G1-2)**	0.6756	(0.6296, 0.7215)	<0.0001
**TNM Stage (III-IV *vs.* I-II)**	0.6655	(0.6189, 0.7121)	<0.0001
**Distant metastasis (Yes *vs.* No)**	0.6947	(0.6313, 0.7580)	<0.0001
**Lymph node metastasis (Yes *vs.* No)**	0.5662	(0.5092, 0.6232)	0.0225
**Tumor size (>1.5cm *vs.* ≤1.5cm)**	0.5993	(0.5483, 0.6503)	0.0002

Abbreviations: AUC: area under the curve, TNM: Tumor node metastasis.
